# Endocast of the Late Triassic (Carnian) dinosaur *Saturnalia tupiniquim*: implications for the evolution of brain tissue in Sauropodomorpha

**DOI:** 10.1038/s41598-017-11737-5

**Published:** 2017-09-20

**Authors:** Mario Bronzati, Oliver W. M. Rauhut, Jonathas S. Bittencourt, Max C. Langer

**Affiliations:** 1 Bayerische Staatssammlung für Paläontologie und Geologie, Richard-Wagner-Straße 10, 80333 Munich, Germany; 2 Department of Earth and Environmental Sciences and GeoBioCenter, Ludwig-Maximilians-Universität, Richard-Wagner-Straße 10, 80333 Munich, Germany; 3 Departamento de Geologia, Universidade Federal de Minas Gerais, Av. Presidente Antônio Carlos 6627, 31270-901 Belo Horizonte, MG Brazil; 4 Labora tório de Paleontologia, FFCLRP, Universidade de São Paulo, Av. Bandeirantes 3900, 14040-901 Ribeirão Preto, SP Brazil

## Abstract

The evolutionary history of dinosaurs might date back to the first stages of the Triassic (c. 250–240 Ma), but the oldest unequivocal records of the group come from Late Triassic (Carnian – c. 230 Ma) rocks of South America. Here, we present the first braincase endocast of a Carnian dinosaur, the sauropodomorph *Saturnalia tupiniquim*, and provide new data regarding the evolution of the floccular and parafloccular lobe of the cerebellum (FFL), which has been extensively discussed in the field of palaeoneurology. Previous studies proposed that the development of a permanent quadrupedal stance was one of the factors leading to the volume reduction of the FFL of sauropods. However, based on the new data for *S*. *tupiniquim* we identified a first moment of FFL volume reduction in non-sauropodan Sauropodomorpha, preceding the acquisition of a fully quadrupedal stance. Analysing variations in FFL volume alongside other morphological changes in the group, we suggest that this reduction is potentially related to the adoption of a more restricted herbivore diet. In this context, the FFL of sauropods might represent a vestigial trait, retained in a reduced version from the bipedal and predatory early sauropodomorphs.

## Introduction

The last two decades have witnessed a rapid development in the world of virtual palaeontology^1^. With the aid of non-destructive computed tomography (CT) techniques, numerous analyses of the internal skull structures of non-avian dinosaurs were carried out. Nevertheless, these studies were mainly based on Jurassic and Cretaceous specimens, whereas the brain and associated soft-tissues of the oldest representatives of the group have never been analysed in detail. The Santa Maria Formation of Brazil, together with the Ischigualasto Formation of Argentina, both Carnian in age (c. 230 Ma), record the oldest unequivocal dinosaurs^2,3^. Cranial remains are not scarce in these strata (e.g. refs^4–7^), but information on the soft tissues associated with the braincase (e.g. brain, inner ear, cranial nerves) are poorly studied (e.g. ref.^8^).

Here, we present the first paleoneurological study of the sauropodomorph *Saturnalia tupiniquim* ^9^. Fossils of *S*. *tupiniquim* come from the Santa Maria Formation in southern Brazil, from a locality commonly known as Cerro da Alemoa or Waldsanga (53°45′ W; 29°40′ S). The taxon is based on three fairly complete specimens [MCP 3844-PV (holotype), 3845-PV, and MCP 3946-PV^9^], but skull elements are only preserved in MCP 3845-PV, including the bones that form the braincase. Given its age and phylogenetic position^10^, *S*. *tupiniquim* is a key-taxon to understand the early evolution of Sauropodomorpha, the lineage that includes the gigantic herbivores of the Mesozoic, the sauropods^10^. Based on new data for *S*. *tupiniquim*, we analyse the evolution of the sauropodomorph endocast in the context of other anatomical transformations and suggest a new scenario for the evolution of the cerebellar neural tissues in the group.

## Results

### Endocast

**Figure 1:**
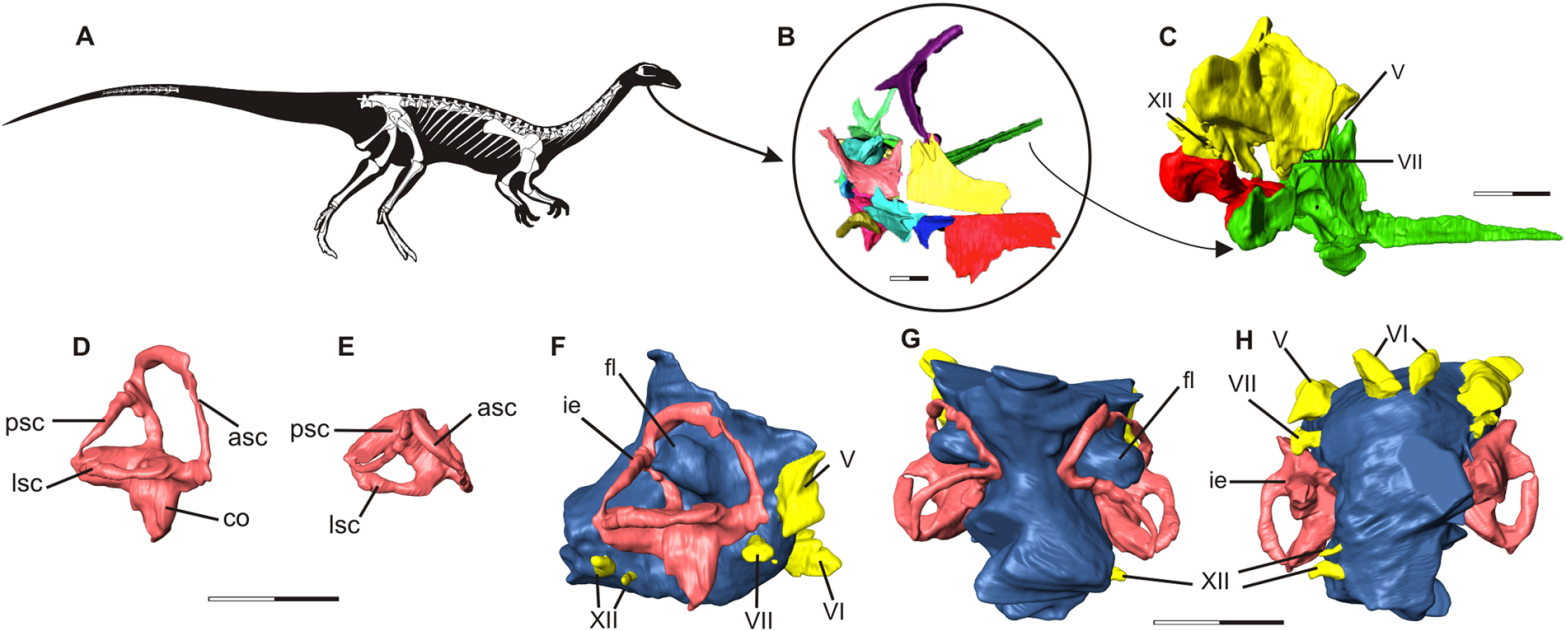
The early sauropodomorph *Saturnalia tupiniquim*. Skeletal reconstruction (**A**). Virtual preparation of cranial bones as preserved inside the matrix (**B**), with braincase highlighted in right lateral (**C**) view. Reconstruction of the soft tissues associated with the braincase: right inner ear in lateral (**D**) and dorsal (**E**) views, and endocast in lateral (**F**), dorsal (**G**), and, ventral (**H**) views. Abbreviations: asc – anterior semicircular canal; co – cochleae; fl – Floccular Fossae Lobe; ie – inner ear; lsc – lateral semicircular canal; psc – posterior semicircular canal; V – trigeminal nerve; VI – abducens nerve; VII – facial nerve; XII hypoglossal nerve. Scale bars = 1 cm.

The endocast of *Saturnalia tupiniquim* presented here (Fig. 1) is based on the reconstruction of the soft tissues associated with the bones of the articulated portion of the preserved braincase, i.e. supraoccipital, otoccipitals (=exoccipital + opisthotic), prootics, parabasisphenoid, and basioccipital (see Supporting Information). Hence, the endocast corresponds to the posterior portion of the brain cavity of *S*. *tupiniquim*, including parts of the hindbrain, such as the cerebellum and medulla oblongata, and cranial nerves V, VI, VII, and XII. The preserved portion of the skull does not include any of the osteological correlates of the fore- or midbrain. Hence, structures such as the olfactory lobes and cranial nerves I to IV are not present in the endocast of *S*. *tupiniquim*.

A flexure in the endocast at the anteroposterior level of the two branches of cranial nerve XII (hypoglossal nerve) is here interpreted as the pontine flexure, with the dorsal margin of the anterior and posterior segments forming angles of approximately 45 degrees to the horizontal plane. In the anterior segment, the dorsal surface of the endocast becomes slightly more vertically orientated, at the same anteroposterior level of large protuberances in the anterolateral part of the endocast. These protuberances are associated with the floccular fossae in the endocranial cavity. It is important to stress that we here consider the floccular fossae as the fossae present in the medial surface of the periotic bones of the skull (*sensu* 11). Accordingly, the protuberances in the matching region of the endocast are here interpreted as corresponding to the neural tissues that filled the floccular fossae. In dinosaurs, these neural tissues might have consisted of the cerebellar flocculus and paraflocculus (see e.g. refs^11,12^), hereafter the FFL (*sensu* 11).

The spatial distribution, number, and general morphology of cranial nerves V, VI, VII, and XII (Fig. 1) in the endocast of *Saturnalia tupiniquim* mostly correspond to that of other dinosaurs (see e.g. refs^13–18^) and non-dinosaurian dinosauriforms^,19^. The foramen associated with the cranial nerve V (trigeminal nerve) is located on the lateral wall of the braincase, anterior to the floccular fossa. In this region of the braincase, there is no evidence of an additional foramen for the lateral branch of the mid-cerebral vein. Thus, the most likely scenario is that this branch exited the braincase via the same aperture as the trigeminal nerve. Accordingly, part of the reconstructed trigeminal nerve in the endocast might also correspond to a portion of the mid-cerebral vein. In contrast to the osteological correlates of other cranial nerves, which pierce the lateral portion of the braincase, the foramina for cranial nerve VI (abducens nerve) are located on its anteroventral surface. Typically, the pituitary fossa is seen ventral to the foramina associated with the cranial nerves VI, but its limits cannot be clearly recognized in the CT-Scan data, and the pituitary gland was not reconstructed. The foramen associated with cranial nerve VII (facial nerve) is completely enclosed by the prootic. It is located ventral to the anterior semi-circular canal of the inner ear and is narrower than those of cranial nerves V and VI. In contrast to the cranial nerves mentioned above, cranial nerve XII (hypoglossal nerve) of *S*. *tupiniquim* was reconstructed only on the right side of the endocast. The two branches of the hypoglossal nerve exited the braincase via independent apertures on the otoccipital on each side of the braincase. As is typical for dinosaurs (e.g. ref.^14^), the posterodorsal foramen is broader than the anteroventral one. The braincase shows a broad aperture posterior to the fenestra vestibule, which might correspond to the metotic foramen (see refs^20–22^); i.e. the exit of cranial nerves IX-XI. However, the path of cranial nerve IX varies greatly among archosaurs, and is not necessarily associated with the metotic foramen^23^. Another possibility is that an extra foramen, the vagal foramen (*sensu* 20), was present in the region of the exoccipital pillar of the ottocipital, representing the path for cranial nerve X, and possibly also for cranial nerve XI in taxa that possess such structure^21^. However, this region of the braincase is not well preserved in MCP 3845-PV, and we chose not to reconstruct cranial nerves IX-XI because of their ambiguous exit places.

The CT data also allowed the reconstruction of the inner ear anatomy of *Saturnalia tupiniquim*. The anterior semi-circular canal (ASC) is approximately 1.5 times higher than wide and the longest of the three canals. Its total length is c. 1.85 the length of the lateral semi-circular canal (LSC), and c. 1.54 the length of the posterior semi-circular canal (PSC). In dorsal view, ASC and PSC diverge from one another, forming an angle of about 80 degrees. Additionally, the portion of PSC between the dorsal limit of the crus commune and the posterior limit of the inner ear is anteriorly curved at an angle of c. 30 degrees; whereas the portion of ASC between its dorsal and anterior limits is straight. The crus commune is caudally curved. At approximately its mid-length, the main axis of the crus is arched at an angle of c. 20 degrees in relation to the vertical axis of the inner ear. The portion of the inner ear ventral to the semi-circular canals is shorter than the dorsoventral length of ASC, but the cochlear duct is not very well preserved, and the ventralmost limit of the cochlea is unclear.

## Discussion

**Figure 2:**
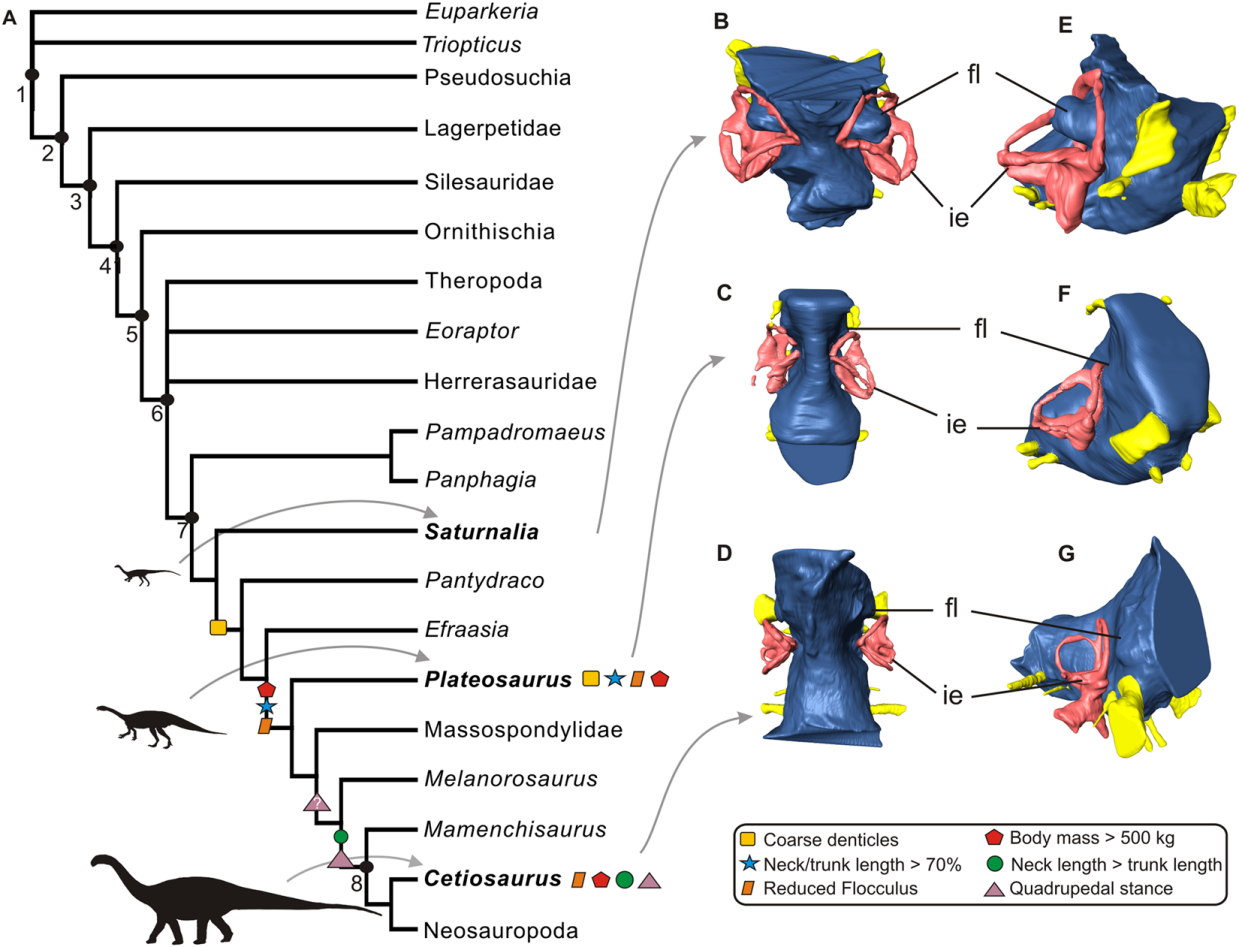
Simplified Archosauriformes phylogeny highlighting character acquisition in Sauropodomorpha (**A**). Endocasts of *Saturnalia tupiniquim* (MCP-3845-PV), *Plateosaurus* (MB.R.5586-1), and a sauropod specimen tentatively reffered to *Cetiosaurus* (OUMNH J13596) in dorsal (**B**,**C**,**D**) and anterolateral (**E**,**F**,**G**) views showing the morphology of the Floccular Fossae Lobe in sauropodomorph dinosaurs. Abbreviations: fl – Floccular Fossae Lobe, ie – inner ear, 1 – Archosauriformes, 2 – Archosauria, 3 – Dinosauromorpha, 4 –Dinosauriformes, 5 – Dinosauria, 6 – Saurischia, 7 – Sauropodomorpha, 8 – Sauropoda.

The floccular fossae lobe (FFL) is part of the systems operating to control eyes, neck, and head movements^11,12,24,25^. As such, it has been investigated in paleoneurological studies of dinosaurs, a group with a wide range of locomotion, feeding behaviour, and ecology (e.g. refs^14,17,25^). In order to trace the evolution of the FFL in sauropodomorph dinosaurs, it is necessary to determine the plesiomorphic and derived conditions of the FFL in the group. Accordingly, the presence of an enlarged protuberance in the region of the endocast corresponding to the FFL, what indicates a large volume of FFL, such as that of *Saturnalia tupiniquim* (Fig. 2), i.e. projecting into the space of the semi-circular canals of the inner ear (this parameter is not employed here in an attempt to capture all the spectrum of size variation in the FFL, but to discriminate between the conditions observed in Carnian sauropodomorphs and sauropods; see below), is also observed in the non-archosaurian archosauriform *Triopticus primus* ^26^ and in non-avian theropods (e.g. 14,25). Based on the size of the floccular fossae in the medial surface of the periotic bones, the condition in the non-archosaurian archosauriform *Euparkeria capensis* ^27^, the non-dinosaurian dinosauriforms *Marasuchus lilloensis* (pers. obs) and *Lewisuchus admixtus* ^19^, and in the silesaurid *Silesaurus opolensis* (pers. obs) mostly likely also correspond to the presence of a large volume of FFL. In this context, the most parsimonious scenario is that *S*. *tupiniquim* retained the plesiomorphic condition for both sauropodomorphs and dinosaurs, and that the small volume of FFL observed in sauropods (e.g. refs^16,17,28^) corresponds to the derived condition within the group. Furthermore, the small volume of FFL of *Plateosaurus engelhardti* indicates that an initial volume reduction of these neural tissues occurred in the early evolutionary history of the group, before the origin of sauropods (Fig. 2).

It is important to stress that previous studies considered that the presence of only a small protuberance in the endocast (associated to the floccular fossae) of sauropods reflected the reduction of the flocculus of the cerebellum^17^. However, as observed in birds, an increase in the volume of the nodulus and the uvula could also cause a protrusion of the flocculus into the fossa, but without an increase in volume of the flocculus itself^12^. Thus, apparent modifications in the volume of the flocculus might be related to transformations in other structures of the brain. Another possibility is that the enlarged protuberance of the endocast related to the floccular fossae, such as that of *Saturnalia tupiniquim* (Fig. 1), might correspond to an artefact of soft-tissue reconstruction for extinct animals. This is because the endocranial cavity can continue to expand after brain maturity^29^ and parts of the fossae could have also housed vasculature tissues^11,12^. Accordingly, any inference on the exact volume of FFL reconstructed in an endocast should be seen with caution^12,29^. However, patterns of anatomical transformations in other parts of the sauropodomorph skeleton and the related shifts in ecology of the taxa belonging to the lineage suggest that the reduction in volume of the portion of the endocast associated to the floccular fossae might indeed correspond to a reduction in the volume of the neural tissues associated with it (i.e. assuming a correlation between the size of the structures and the amount of neural information they process), rather than representing only an artefact related to the presence of vasculature tissue in this region.

The apparent smaller volume of FFL observed in sauropod endocasts has been associated with their quadrupedal stance^17,28,30^. The rationale behind this correlation lies in the requirement of a more refined balance control in bipedalism than in quadrupedalism, in a scenario where balance is coordinated by a neurological chain that involves the FFL and the inner ear^14,24^. However, analyses of endocasts of other dinosaurs indicate a much more complex scenario. *Saturnalia tupiniquim* is a facultative biped^31,32^ with the condition of the FFL differing from the one of the also facultative or even obligate biped sauropodomorph *Plateosaurus engelhardti* ^33^, in which the reconstructed FFL in the endocast do not project into the space between the semicircular canals. Among bipedal theropods, taxa such as therizinosaurids^25^ and *Tyranossaurus rex* ^14^ exhibit reconstructed FFL that project into the space of the inner ear, but a great variation is observed among these taxa, with the FFL volume in the former being much greater than in the latter. Furthermore, some quadrupedal ornithischians^34^ also have the reconstructed FFL projecting into the space of the semicircular canals. Thus, the corresponding variation in volume of this structure, not only between bipedal and quadrupedal forms, but also within taxa with the same pattern of locomotion, indicates that the variation detected for the FFL in the endocast of dinosaurs is not solely related to locomotion. Indeed, it has been demonstrated that the association of a specific FFL volume to a single biological condition might be misleading^11^.

Tracing transformations in the endocast of sauropodomorphs alongside other osteological modifications indicate that the change in feeding behaviour, from active predators to herbivores or scavenger omnivores, is one of the factors that can be potentially associated with the reduction of the floccular and parafloccular lobes. Given the neurological link of these neural tissues with control mechanisms, such as the vestibulo ocular reflex (VOR) and vestibulo collic reflex (VCR), it has been suggested that a well-developed flocculus could be linked to active predation in dinosaurs^25^. Indeed, an increase in VOR capacity leads to an enhanced gaze stabilization^11,24,29^, which enables animals to better focus on prey while coordinating the neck and skull during fast movements. On the other hand, the VCR is linked to the control of head and neck movements, for which the nodulus and the uvula participate in the neural processing of linear head acceleration signals^35^. In this case, the enhancement of VCR capacity might be crucial to pursue effective attacks on small and elusive prey.

*Saturnalia tupiniquim* has been originally interpreted as a herbivorous animal^9^, mostly taking into account its sauropodomorph affinities, which was hitherto interpreted as a typically herbivore clade, rather than based on specific aspects of its anatomy. However, its tooth morphology (see Supporting Information) shows features also found in dinosaurs able to use food sources other than plants^36–38^. Tooth crowns are recurved and possess small serrations that are perpendicular to the carina, as is typical for faunivorous taxa^37–39^. Hence, based on tooth morphology alone, faunivorous or omnivorous diet reconstructions are equally likely for *S*. *tupiniquim*, but its large volume of FFL provides potential additional evidence of its predatory behaviour (see above). In contrast to the oldest Carnian sauropodomorphs, other Late Triassic members of the group, such as *Efraasia minor* and *Plateosaurus engelhardti*, possess lanceolate teeth with coarse denticles, features usually associated with a diet mainly based on plants^39^. These taxa might eventually have complement their diet with scavenging^40^, a less “active” means of gathering animal food. Moreover, the first steps towards body size increase in Sauropodomorpha happened in the least inclusive clade including *P*. *engelhardti* and sauropods^41–43^. The increase in body size has been demonstrated to have been crucial for the evolution of a fully herbivorous diet in Sauropodomorpha^42,43^. In this context, when characters of hard and soft-tissues are mapped onto a phylogeny, the loss of neurological traits potentially related to an efficient predation (i.e. FFL reduction) is detected alongside modifications associated with a more obligate herbivorous diet (Fig. 2), in a clade including taxa such as *P*. *engelhardti* and sauropods, but not *S*. *tupiniquim* and other faunivore/omnivore Carnian taxa.

Another factor that might be associated with the variation in the volume of the FFL in sauropodomorphs is the elongation of the neck in this lineage. We estimated that neck length of S. tupiniquim accounts for c. 0.56–0.60 of the trunk (see Supporting Information). This is only slightly elongated if compared with early dinosauriforms such as *Marasuchus* and *Silesaurus*, in which this proportion is not greater than 0.5^44,45^. In early dinosaurs such as *Eoraptor* and *Heterodontosaurus*, the neck/trunk relative length varies between c. 0.5 and c. 0.55^46,47^. A more significant cervical elongation among sauropodomorphs is firstly observed in the minimal clade including *Plateosaurus* (neck length/trunk length c. 0.75) and sauropods, which typically  exhibit necks longer than the trunk^43^. In this case, a reduction in FFL volume could be the result of the reduction in the neural processing related to the VOR. This is because in dinosaurs with elongated necks, such as *Plateosaurus* and sauropods, the early detection of head movement is likely to be less critical for balance because the head is further decoupled from the trunk. Nevertheless, the presence of an elongated neck could also lead to an increase in neural processing of VCR, which controls cervical posture^14,^ ^24^. In this context, neck elongation seems to have also played an important role in FFL evolution in sauropodomorphs, but with opposite effects for VOR and VCR.

In conclusion, a significant reduction of the FFL in sauropodomorphs in the last stages of the Triassic (i.e. Norian) seems to be associated with anatomical modifications related to the adoption of a herbivorous diet. Given the role that the FFL have in the visual coordination and head/neck movements, this suggests that the transition to herbivory also involved neurological modifications in Sauropodomorpha (Fig. 2). Nevertheless, two caveats should be noted. First, our delimitation of what represents greater volumes of FFL (based on the projection of the corresponding portion of the endocast into the space of the inner ear – see Figs 1 and 2) fails to capture all possible variations in volume of the neural tissues. However, a reduction in the volume of the FFL (i.e. the corresponding portion of the endocast not entering the inner ear space) is already observed in *Plateosaurus engelhardti*, indicating that an initial reduction took place among bipedal sauropodomorphs^33^, before the evolution of a fully quadrupedal stance. Second, it is worth stressing that many evolutionary drivers (i.e. evolution of herbivory, adoption of a quadrupedal stance, elongation of the neck) might have played a role in the evolution of the FFL in non-sauropodan sauropodomorphs. In this case, only a throughout investigation including a larger sample of endoscasts of non-sauropodan sauropodomorph taxa alongside studies on the evolutionary drivers in extant taxa (e.g. refs^11,12^) will be able to clarify the evolution of the FFL in the lineage. Finally, making inferences on the lifestyle of extinct taxa using a single criterion can be misleading^11,^ ^12^. Form/function correlations should be very carefully made^11^, and other parameters (historical and ahistorical) should be taken into account when inferring the ecology of extinct taxa^48^. In this sense, when analysed alongside other anatomical features, the cranial soft tissues reconstructed for *Saturnalia tupiniquim* are consistent with the interpretation that early sauropodomorphs had a predatory behaviour^37^.

## Material and Methods

### Specimen and CT-Scan

Computed tomography data was used to produce a virtual model of the soft tissues associated with the braincase of *Saturnalia tupiniquim*. The specimen was scanned at the Zoologische Staatsammlung München (Bavaria State Collection of Zoology, Munich, Germany) in a Nanotom Scan (GE Sensing & Inspection Technologies GmbH, Wunstorf Germany) using the following parameters: Voltage: 100 Kv; Current: 130 μA; 3.1 μm voxel size. 1,440 slices were generated, which were downsampled by half and segmented in the software Amira (version 5.3.3, Visage Imaging, Berlin, Germany). The CT-Scan data show that otoccipital (= exoccipital + opisthotic), parabasisphenoid (= parasphenoid + basisphenoid), basioccipital, and supraoccipital are preserved in articulation inside the matrix (see Supporting Information), allowing a precise reconstruction of the posterior portion of the endocranial cavity

### Phylogeny of Sauropodomorpha

In order to trace morphological transformations and major changes in the feeding behaviour along sauropodomorph evolution, we constructed an informal supertree based on the results of the most recent phylogenetic analyses for the group and its closest relatives (e.g. refs^7,22,26,36,37,49–53^). The discovery of new taxa, such as *Panphagia protos* ^54^, *Chromogisaurus novasi* ^50^, *Pampadromaeus barbarenai* ^6^, and *Buriolestes schultzi* ^37^, along with the reassessment of the phylogenetic position of *Eoraptor lunensis* as a sauropodomorph^7,37^, provided new data and interpretations, but the relationships of the earliest sauropodomorphs from the Carnian Santa Maria and Ischigualasto formations are still uncertain (see e.g. ref.^2^). Nevertheless, the nesting of *Saturnalia tupiniquim* within sauropodomorphs has been consistently confirmed by independent studies^7,22,26,36,37,49–53^. Regarding other non-sauropod sauropodomorphs, there is a growing consensus that no clade congregates all (nor most) taxa classically treated as ‘Prosauropoda’ to the exclusion of Sauropoda. Instead, these taxa have recently been found to represent a paraphyletic array in relation to Sauropoda^22,50–53^.

## Electronic supplementary material

Supplementary Information
