# Supplementary material for: Endocast of the Late Triassic (Carnian) dinosaur Saturnalia tupiniquim: implications for the evolution of brain tissue in Sauropodomorpha
Source: Sci Rep. 2017 Sep 20;7:11931. doi: 10.1038/s41598-017-11737-5 (PMC5607302; doi:10.1038/s41598-017-11737-5)
Supplement: Supplementary file 1 — Supplementary Information [file 41598_2017_11737_MOESM1_ESM.pdf]

Supporting Online Material for

Endocast of the Late Triassic (Carnian) dinosaur *Saturnalia tupiniquim*: implications for the evolution of neurological tissues in Sauropodomorpha

Mario Bronzati[1,2,*], Oliver W. M. Rauhut[1,2], Jonathas S. Bittencourt[3], Max. C. Langer[4]

[1] Bayerische Staatssammlung für Paläontologie und Geologie, Richard-Wagner-Straße 10, 80333 Munich, Germany.

[2] Department of Earth and Environmental Sciences and GeoBioCenter, Ludwig-Maximilians-Universität, Richard-Wagner-Straße 10, 80333 Munich, Germany.

[3] Departamento de Geologia, Universidade Federal de Minas Gerais, Av. Presidente Antônio Carlos 6670, 31270-901, Belo Horizonte MG, Brazil

[4] Laboratório de Paleontologia, FFCLRP, Universidade de São Paulo, Av. Bandeirantes 3900, 14040-901, Ribeirão Preto SP Brazil.

*To whom correspondence should be addressed.
E-mail: m.bronzati@lrz.uni-muenchen.de; mariobronzati@gmail.com

SUPPLEMENTARY INFORMATION

1. Institutional abbreviations

GPIT, Institut und Museum für Geologie und Paläontologie, Universität Tübingen, Tübingen, Germany; **MB**, Museum für Naturkunde, Berlin, Germany; **MCP**, Museu de Ciências e Tecnologia, Pontificia Universidade Católica do Rio Grande do Sul, Porto Alegre, Brazil; **OUMNH**, Oxford University Museum of Natural History, Oxford, UK; **SMNS**, Staatliches Museum für Naturkunde, Stuttgart, Germany; **ULBRA-PV**, Museu de Ciências Naturais, Universidade Luterana do Brasil, Canoas.

2. Specimen MCP-3845-PV of *Saturnalia tupiniquim*

Saturnalia tupiniquim is known from three specimens: MCP 3844 (holotype) - 3846-PV (see Langer, 2003 for more details). The fossils come from the Carnian Santa Maria Formation, in southern Brazil, from a locality commonly known as Cerro da Alemoa or Waldsanga (53°45’ W; 29°40’ S). Langer et al. (1999) provided a very preliminary description of *S. tupiniquim*, but more detailed descriptions of the pelvic (Langer, 2003) and scapular (Langer et al. 2007) girdles and limbs were provided later. The braincase is only preserved in MCP-3845-PV (Figure S1), and was never studied in detail. Its full description is under preparation, and will be presented elsewhere.

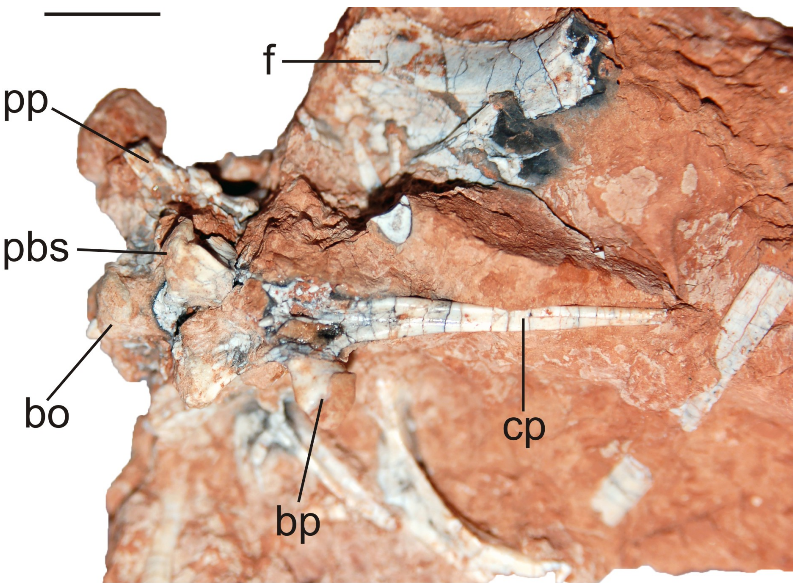

Figure S1: *Saturnalia tupiniquim* (MCP-3845-PV). Block containing the braincase and other skull elements. bo – basicoccipital; bp – basipterygoid process; cp – cultriform process of the parabasisphenoid; f – frontal; pbs – parabasisphenoid; pp – paroccipital process of the otoccipital. (scale bar = 10mm)

The block containing the skull of MCP-3845-PV has multiple fractures, hampering its mechanical preparation. Computed tomography was, therefore, employed in order to access the braincase osteology of *Saturnalia tupiniquim* (Figure S2).

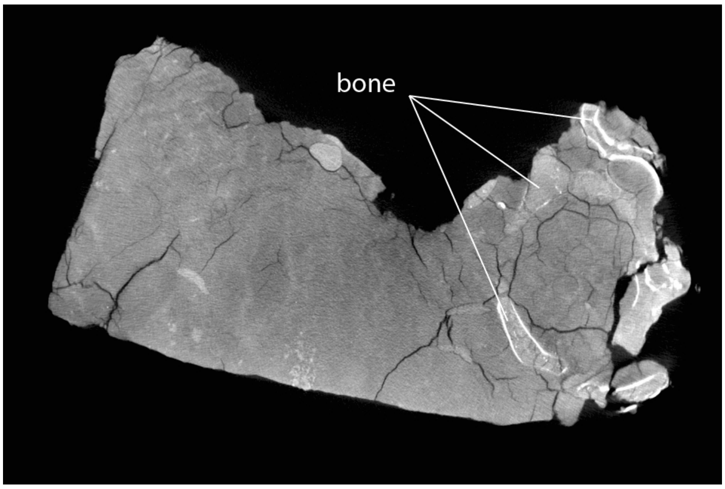

Figure S2: *Saturnalia tupiniquim* (MCP-3845-PV). Example of slice obtained from the Computed Tomographic. The contrast between bones and matrix allows a precise reconstruction of the osteology and soft-tissue anatomy of the braincase.

The CT-Scan data show that otoccipital (= exoccipital + opisthotic *sensu* Sampson & Witmer, 2007), parabasisphenoid, basioccipital, and supraoccipital are preserved in articulation inside the matrix (Figure S3), allowing a detailed reconstruction of the posterior portion of the endocranial cavity (see Main Document).

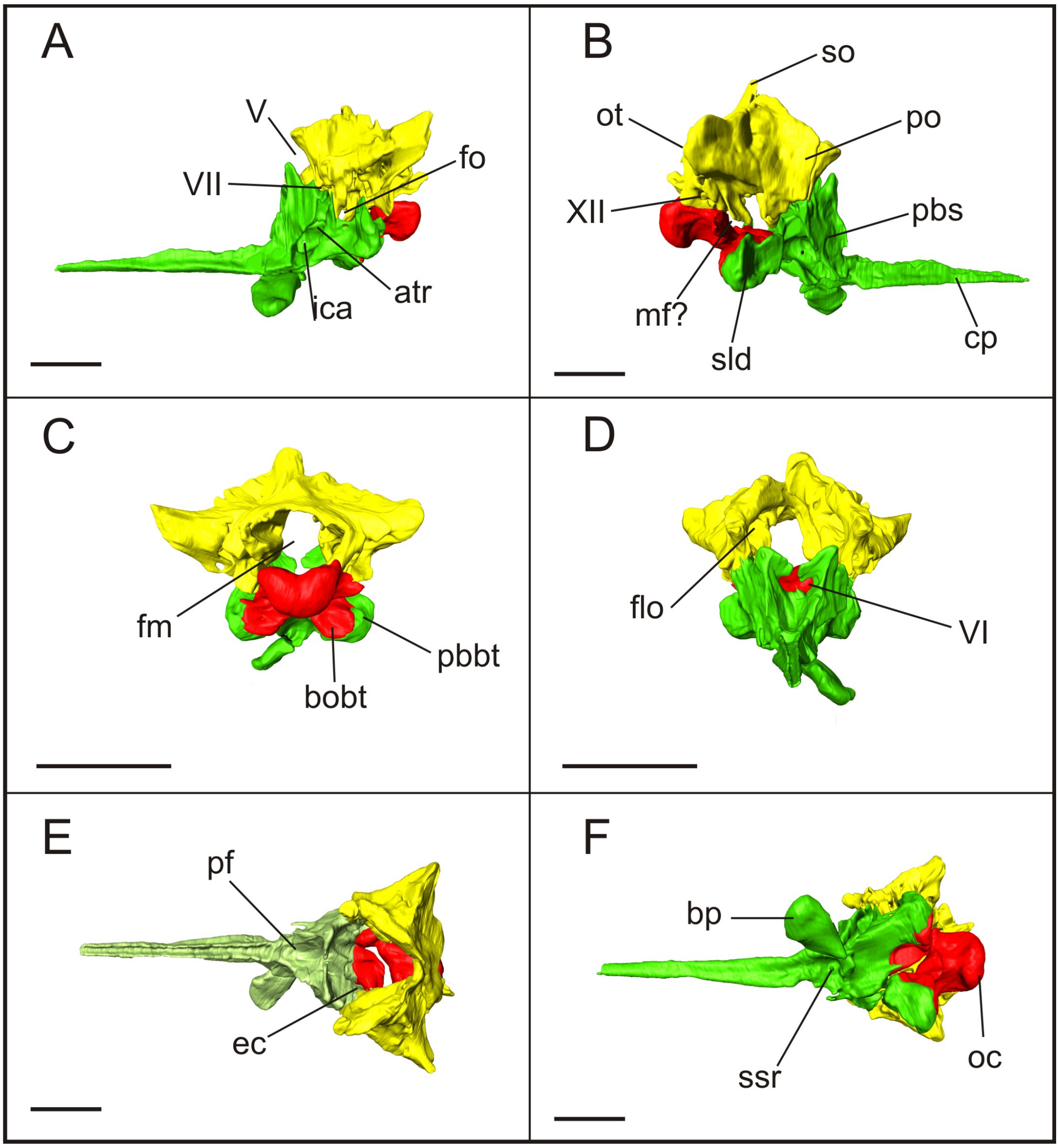

Figure S3: *Saturnalia tupiniquim* (MCP-3845-PV). Results of the braincase segmentation in left lateral (A), right lateral (B), occipital (C), anterior (D), dorsal (E), and ventral (F) views. atr – anterior tympanic recess; bobt - basioccipital component of the basal tubera; cp – cultriform process of the parabasisphenoid; ec –

endocranial cavity; flo – flocculus of the cerebellum; fm – foramen magnum; fo – fenestra ovalis; ica – internal carotid artery; mf – metotic foramen; ot – otoccipital; pbbt – parabasisphenoid component of the basal tubera; pbs – parabasisphenoid; pf – pituitary fossa; po – prootic; sld – semi-lunar depression; so – supraoccipital; ssr – subsellar recess; V – trigeminal nerve; VI – abducens nerve; VII – facial nerve; XII – hypoglossal nerve. (scale bars = 10 mm).

3. Dentition of *Saturnalia tupiniquim* and other sauropodomorphs

Inferences on the diet of the earliest dinosaurs have been made mostly based on their tooth morphology, on a form-function correlation approach (Barrett & Rayfield, 2006). Yet, a complete separation between an omnivore and a facultative herbivore diet is usually not possible solely on the basis of tooth morphology (Barrett, 2000; Barrett & Upchurch, 2007). Nevertheless, the earliest Sauropodomorpha exhibit tooth traits that are related to a carnivorous diet, which are not seen in later members of the lineage (Figure S4 - see Main text for details).

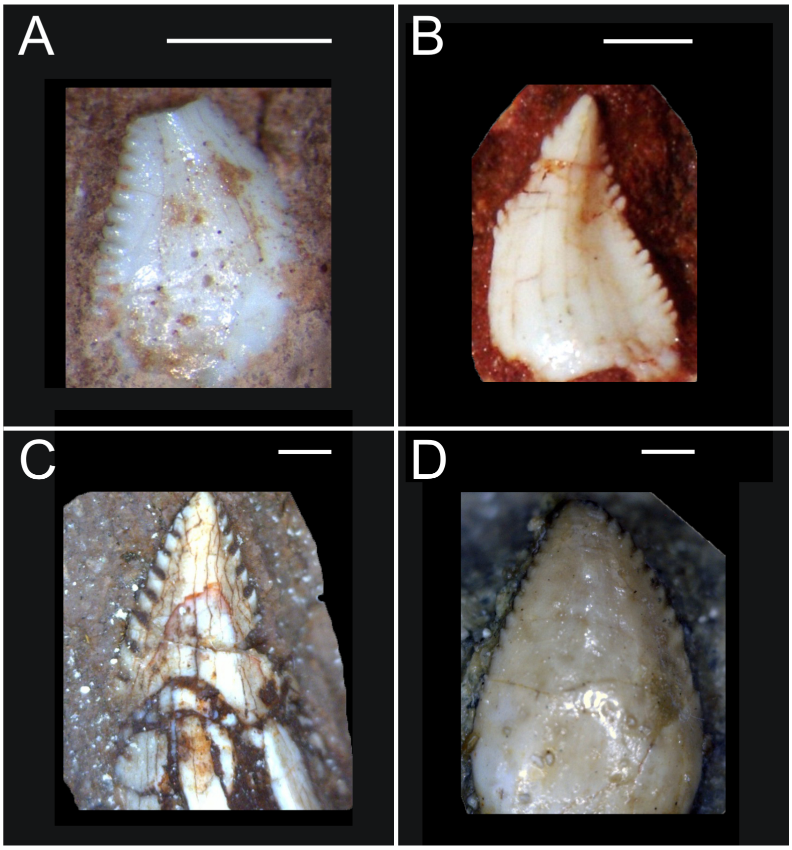

Figure S4: Mandibulary teeth of the sauropodomorph dinosaurs *Saturnalia tupiniquim* (A), *Pampadromaeus barbarenai* – ULBRA PVT 016 (B), *Efraasia minor* – SMNS 12684 (C); *Plateosaurus gracilis* – GPIT 18318a (D).

4. Neck morphology of *Saturnalia tupiniquim*

One paratype of *Saturnalia tupiniquim* (MCP-3845-PV) preserved 22 semi-articulated presacral vertebrae; the atlantal intercentrum, plus neural arch, and the caudalmost trunk vertebrae are missing. A conspicuous morphological transition between presacral vertebrae 9 and 10, including a rectangular rather than parallelogram centrum shape and a larger area for the tuberculum attachment in the vertebra 10, suggests that *S. tupiniquim* has 9 "typical" cervical vertebrae (Figure S5). Indeed, the neck/trunk transition in early dinosaurs with more complete vertebral series available, e.g. *Eoraptor*, *Staurikosaurus*, *Coelophysis*, *Plateosaurus*, *Heterodontosaurus* (Santa Luca, 1980; Colbert, 1989; Galton and Upchurch, 2004; Bittencourt and Kellner,

2009; Rinehart, Lucas, Heckert, Spielmann and Celesky, 2009; Sereno, Martínez and Alcober, 2012), is positioned at presacrals 9 or 10. In the absence of further evidence concerning the exact transition point between the neck and trunk of *S. tupiniquim* (e.g. articulated ribs and scapular girdle), we estimated its neck length alternatively with 9 or 10 vertebrae. Except for *Heterodontosaurus*, the above-mentioned dinosaurs are thought to possess 15 trunk ("dorsal") vertebrae, which is assumed herein for *S. tupiniquim*. The presacral column of *S. tupiniquim* is thus reconstructed as having 24 or 25 vertebrae.

We estimated that the neck of *S. tupiniquim* accounts for c. 56-60% of the trunk (Tables S1–S2). This is slightly elongated if compared with early dinosauriforms such as *Marasuchus* and *Silesaurus* (Sereno and Arcucci, 1994; Piechowsky and Dzik, 2010), in which this proportion is not greater than 50%. In several early dinosaurs, e.g. *Eoraptor*, *Heterodontosaurus* (Santa Luca, 1980; Sereno et al., 2012), the neck/trunk relative length varies between 50–55%. A more significant cervical elongation is seen in early neotheropods, e.g. *Coelophysis* (88%), and firstly in *Plateosaurus* (75%) among sauropodomorphs (Rauhut, Fechner, Remes and Reis, 2011). The neck elongation in *S. tupiniquim* is intermediate between that of early saurischians and plateosaurians (i.e. members of the clade Plateosauria). The paucity of anatomical data for other early sauropodomorphs (i.e., *Panphagia*, *Pampadromaeus*, and *Chromogisaurus*), or even for taxa close to plateosaurians, hampers an accurate assessment of the initial pace of cervical elongation within sauropodomorphs.

Table S1 – Ventral length (in mm) of the presacral centra of *Saturnalia tupiniquim* (MCP-3845-PV)

ps2*	ps3	ps4	ps5	ps6	ps7	ps8	ps9	ps10†	ps11	ps12
**22**	**22.47**	**--**	**23.88**	**23.13**	**22.48**	**20.25**	**18.3**	**17.67**	**16.79**	**17.9**

ps13	ps14	ps15	ps16	ps17	ps18	ps19	ps20	ps21	ps22	ps23
**20.2**	**--**	**21.9**	**22.85**	**23.76**	**23.72**	**23.75**	**23.29**	**24.26**	**22.88**	**--**

*Axis (including axial intercentrum)
†Last neck vertebra **or** first trunk vertebra
-- Incomplete centrum

Table S2 – Estimation of the neck and trunk length (in mm) of *Saturnalia tupiniquim* (MCP-3845-PV)

	Neck lenght (min.-max.)*	Trunk length (min.-max.)*	Neck-trunk ratio (min.-max)
9 neck vertebrae	**182.3–183.7**	**324.9–326.6**	**55.8–56.5%**
10 neck vertebrae	**199–200.4**	**330.1–331.8**	**60–60.7%**

*ps4 and ps14 estimated with basis on the adjacent vertebrae (min.-max.)
*atlas length corresponds to 1/3 of the axis
*values for the caudalmost presacrals are based on ps22.

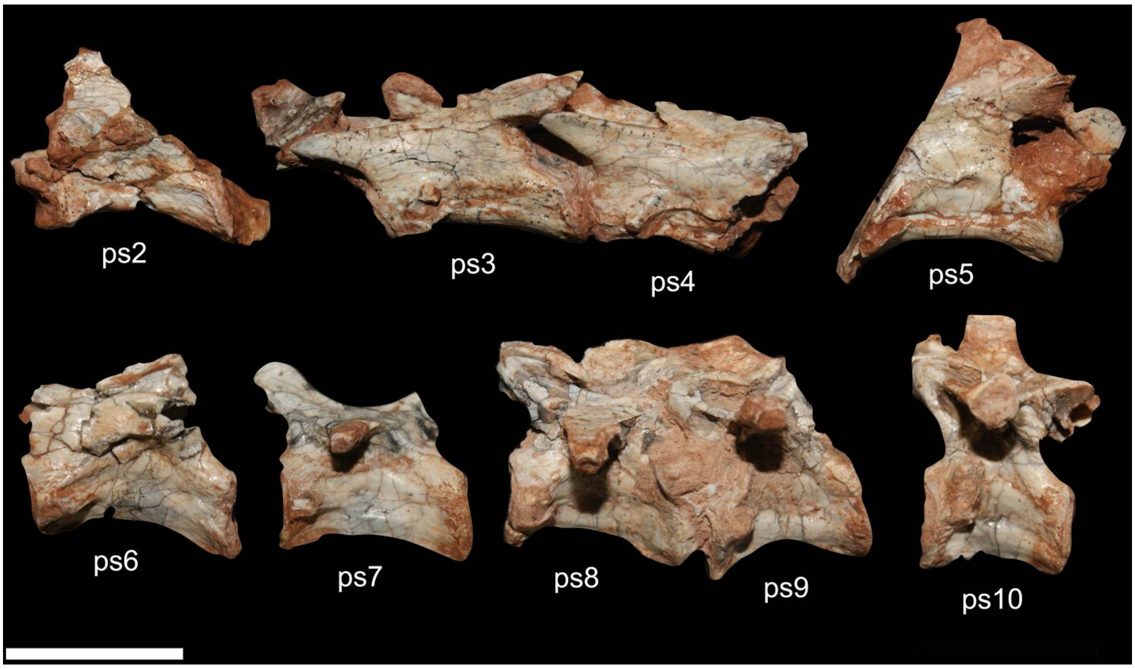

Figure S5 – *Saturnalia tupiniquim* (MCP-3845-PV), presacral vertebrae from 2 (axis) to 10. Scale bar = 20 mm.

5. Additional References

Barrett, P. M. 2000. Prosauropods and iguanas: speculation on the diets of extinct reptiles, In: (ed. H.-D. Sues) *Evolution of Herbivory in Terrestrial Vertebrates: Perspectives from the Fossil Record*. Cambridge University Press: Cambridge , pp. 42–78 .

Barrett, P. M., Upchurch, P. 2007. The evolution of feeding mechanisms in early sauropodomorph dinosaurs. *Special Papers in Palaeontology,* 77: 91–112.

Bittencourt, J. S. & Kellner, A. W. A. 2009. The anatomy and phylogenetic position of the Triassic dinosaur *Staurikosaurus pricei* Colbert, 1970. *Zootaxa*, 2079, 1–56.

Colbert, E. H. 1989. The Triassic dinosaur *Coelophysis*. Museum of Northern Arizona Bulletin, 57, 1–160.

Galton, P. M., Upchurch, P. 2004. Prosauropoda. In. (eds. D. B. Weishampel, P. Dodson and H. Osmólska) *The Dinosauria, second edition*University of California Press, Berkley. pp. 232–258

Langer, M. C., Abdala, F., Richter, M., Benton, M. J. 1999. A sauropodomorph dinosaur from the Upper Triassic (Carnian) of southern Brazil. *Earth & Planetary Sciences*, 329, 511-517.

Langer, M. C. 2003. The sacral and pelvic anatomy of the stem-sauropodomorph *Saturnalia tupiniquim* (Late Triassic, Brazil). *Paleobios* 23(2): 1–40.

Langer, M. C., França, M.A.G., & Gabriel, S. 2007. The pectoral girdle and forelimb anatomy of the stem sauropodomorph *Saturnalia tupiniquim* (Late Triassic, Brazil). In. (eds. P. M. Barrett and D. J. Batten) *Evolution and palaeobiology of early sauropodomorph dinosaurs. Special Papers in Palaeontology*, 77, 113–137.

Piechowsky, R. & Dzik, J. 2010. The axial skeleton of Silesaurus opolensis. Journal of Vertebrate Paleontology, 30, 1127–1141.

Rauhut, O. W. M., Fechner, R., Remes, K. & Reis, K. 2011. How to get big in the Mesozoic: the evolution of the sauropodomorph body plan. Pp. 119-149 in N. Klein, K. Remes, C. T. Gee & M. Sander (eds) Biology of the sauropod dinosaurs: understanding the life of giants, Indiana University Press, Bloomington.

Rinehart, L. F., Lucas, S. G., Heckert, A. B., Spielmann, J. A. & Celesky, M. D. 2009. The paleobiology of Coelophysis bauri (Cope) from the Upper Triassic (Apchean Whitaker quarry, New Mexico), with detailed analysis of a single quarry block. Bulletin of the New Mexico Museum of Natural History and Science, 45, 1–260.

Sampson, S. D., Witmer, L. M. 2007. Craniofacial anatomy of *Majungasaurus crenatissimus* (Theropoda: Abelisauridae) from the Late Cretaceous of Madagascar. *Journal of Vertebrate Paleontology*, 27:32 – 102.

Santa Luca, A. P. 1980. The postcranial skeleton of Heterodontosaurus tucki (Reptilia, Ornithischia) from the Stormberg of South Africa. Annals of the South African Museum, 79, 159–211.

Sereno, P. C. & Arcucci, A. B. 1994. Dinosaurian precursors from the Middle Triassic of Argentina: Marasuchus lilloensis, n. gen. Journal of Vertebrate Paleontology, 14, 53–73.
Sereno, P. C., Martínez, R. N. & Alcober, O. 2012. Osteology of Eoraptor lunensis (Dinosauria, Sauropodomorpha). Journal of Vertebrate Paleontology, 32:sup 1, 83-179.
